# Low-Temperature Hydrotreatment of C4/C5 Fractions Using a Dual-Metal-Loaded Composite Oxide Catalyst

**DOI:** 10.3390/nano14231934

**Published:** 2024-11-30

**Authors:** Zhou Du, Renyi Li, Zhenghui Shen, Xiao Hai, Ruqiang Zou

**Affiliations:** 1 Beijing Key Laboratory for Theory and Technology of Advanced Battery Materials, School of Materials Science and Engineering, Peking University, No. 5 Yiheyuan Road, Haidian District, Beijing 100871, China; 2101120084@stu.pku.edu.cn (Z.D.); renyili@bit.edu.cn (R.L.); zhshen@pku.edu.cn (Z.S.); xiaohai@pku.edu.cn (X.H.); 2 Yanshan Branch, SINOPEC (Beijing) Research Institute of Chemical Industry Co., Ltd., No. 15 Fenghuangting Road, Fangshan District, Beijing 102500, China

**Keywords:** hydrotreating catalysts, low-temperature activity, non-noble metal catalyst, C4 and C5 fractions, reducing the energy consumption

## Abstract

C4 and C5 fractions are significant by-products in the ethylene industry, with considerable research and economic potential when processed through hydrogenation technology to enhance their value. This study explored the development of hydrotreating catalysts using composite oxides as carriers, specifically enhancing low-temperature performance by incorporating electronic promoters and employing specialized surface modification techniques. This approach enabled the synthesis of non-noble metal hydrogenation catalysts supported on Al_2_O_3_–TiO_2_ composite oxides. The catalysts were characterized using various techniques, including X-ray diffraction, N_2_ adsorption-desorption, scanning electron microscopy, X-ray photoelectron spectroscopy, ammonia temperature-programmed desorption, infrared spectroscopy, and transmission electron microscopy. Mo–Ni/Al_2_O_3_–TiO_2_ catalysts were optimized for low-temperature hydrotreating of C4 and C5 fractions, demonstrating stable performance at inlet temperatures far below those typically required. This finding enables a shift from traditional gas-phase to gas–liquid two-phase reactions, eliminating the need for high-pressure steam in industrial settings. As a result, energy consumption is reduced, and operational stability is significantly improved.

## 1. Introduction

C4 and C5 fractions are vital resources in the development of comprehensive industrial utilization of petrochemicals [1]. In the steam cracking process for ethylene production, C4 and C5 yield represent approximately 15% and 35% of the ethylene yield, respectively. By the end of 2022, China’s ethylene production capacity had reached 49.3 million tons [2], generating around 7.4 million tons per year of C4 and 17.3 million tons per year of C5 as by-products. Efficient utilization of these substantial C4 and C5 resources is critically needed. Hydrotreatment technology offers a promising solution for processing C4 and C5 fractions, enhancing the value of raw materials while addressing environmental concerns [3,4]. Due to their lower boiling points, C4 and C5 fractions vaporize easily at atmospheric pressure, and the by-products contain mostly mono-olefins, di-olefins, and alkynes. After the extraction of di-olefins and other hydrocarbons, the remaining alkynes, unsaturated hydrocarbons, and impurities become concentrated, leading to polymerization and gum formation, which complicates their application for other purposes. This results in underutilization of these resources, with a considerable portion being burned as fuel.

With the continued growth in ethylene production capacity, the effective utilization of light hydrocarbon resources has become increasingly prominent. Converting C4 and C5 alkanes through full hydrotreatment for use as high-quality cracking feedstock provides both environmental and economic benefits. Therefore, full hydrotreatment represents a crucial pathway for the efficient utilization of C4 and C5 by-products from cracking. Hydrotreatment catalysts for C4 and C5 are primarily classified into two categories: noble metal catalysts, such as palladium (Pd), platinum (Pt), and silver (Ag), and non-noble metal catalysts, including copper (Cu) and nickel (Ni) [5]. Noble metals like Pd exhibit high catalytic activity and selectivity, and the selective hydrotreatment processes designed for them continue to dominate the field [6]. However, the high cost of noble metals, their susceptibility to poisoning during reactions, and their short regeneration cycles limit the broader application of Pd catalysts. In contrast, Ni-based catalysts demonstrate strong resistance to impurities in catalytic hydrotreatment [7], making them less susceptible to poisoning. This characteristic allows for an extended lifespan of the catalysts, and due to the low price of Ni, it is considered an ideal active component for selective hydrotreatment processes [8,9].

In industrial applications, cost-effective molybdenum–nickel catalysts are widely used in hydrotreatment reactions [10]. However, current research and industrial technologies require high reaction temperatures for olefin hydrotreatment, typically above 200℃. For instance, a study by Niu et al. [11] suggests that the primary hydrotreatment of olefins occurs at 230 °C. As a result, hydrotreatment units in factories require high-pressure steam (4.0 MPa) as a heat source, leading to high energy consumption. To address these demands, manufacturers seek a non-noble metal hydrogenation catalyst with higher low-temperature activity, low cost, and strong resistance to impurities, aiming to reduce the inlet temperature and thereby lower energy consumption. Preliminary experiments conducted in the laboratory using optimized hydrogenation catalysts have successfully reduced the inlet temperature of the reactor to below 140 °C, eliminating the need for heated high-pressure steam and significantly reducing energy consumption and operating costs. Additionally, the lower temperatures minimize side reactions, resulting in improved operational stability of the equipment. This leads to energy savings, emission reduction, cost efficiency, and increased productivity, delivering notable economic benefits. Consequently, further advancements in low-temperature-active catalysts and the development of related technologies will enhance the effective utilization of C4 and C5 resources.

## 2. Results and Discussion

### 2.1. Catalyst Synthesis and Characterization

The preparation process for the selected Al_2_O_3_–TiO_2_ composite oxide support is shown in Figure S1. The supported Mo–Ni/Al_2_O_3_–TiO_2_ hydrogenation catalyst was synthesized using the equal volume impregnation method. The initial step in catalyst preparation is to determine the water absorption rate of the support. This procedure involves taking an appropriate amount of the support material (mass A), soaking it in deionized water for 15 min, then using filter paper to remove excess water until the support is completely moistened. The support is then weighed again (mass B). The water absorption of the support is calculated as the weight difference (B − A), and the water absorption rate (R) can be determined according to the following formula:

$$R=\frac{B-A}{A}\times 100\%$$

The catalyst preparation procedure is as follows. The synthesized Al_2_O_3_–TiO_2_ composite support is initially calcined at 550 °C for 4 h. Subsequently, an ammonium molybdate impregnation solution of specific concentration is prepared using a volumetric flask, adjusted according to the water absorption rate of the support. The support is then impregnated with this solution for a designated period, followed by overnight drying and a secondary calcination at 550 °C for 4 h. In the second stage, the support is impregnated with a nickel nitrate solution in two equal portions, each following the same procedure as described above, to produce the hydrogenation catalyst Mo–Ni/Al_2_O_3_–TiO_2_ (BY-6H), containing active components with MoO_3_ and NiO loading of 10–20 wt.% and 4–15 wt.%, respectively.

**Figure 1:**
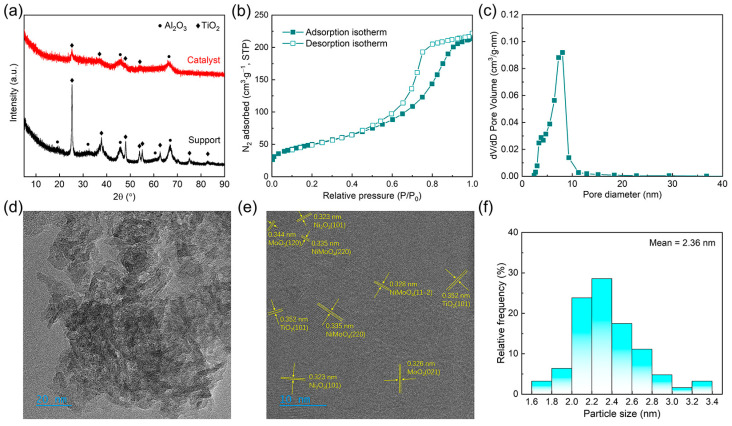
Structure characterization of the prepared Mo–Ni/Al_2_O_3_–TiO_2_ catalysts. (**a**) XRD patterns. (**b**) N_2_ adsorption–desorption isotherm. (**c**) Pore size distribution. (**d**) TEM and (**e**) HRTEM images. (**f**) Particle size distribution.

Figure 1a displays the XRD patterns of both the support and the catalyst. Characteristic diffraction peaks of γ-Al_2_O_3_ are observed at 2θ = 45.6° and 66.5°, indicating that the Al_2_O_3_ component in the catalyst exists in the γ-Al_2_O_3_ phase. A characteristic diffraction peak of anatase is observed at 2θ = 25.4°, confirming the presence of the TiO_2_ component in the form of an anatase phase within the catalyst. In the XRD pattern of the Mo–Ni/Al_2_O_3_–TiO_2_ catalyst, the anatase phase diffraction peak appears at 2θ = 25.3°, with γ-Al_2_ O_3_ peaks observed at 2θ = 45.6° and 66.3°, indicating that the primary structure of the support is retained after loading with Mo and Ni metals. Notably, the absence of characteristic diffraction peaks associated with NiO, Ni_2_O_3_, and molybdenum species suggests a high degree of dispersion of the active nickel and molybdenum species on the surface of the catalyst [12,13]. This uniform dispersion is beneficial for catalytic performance, as it enhances the accessibility of active sites, which may improve the overall catalytic activity and selectivity of the catalyst.

The N_2_ adsorption–desorption isotherm and pore size distribution for the BY-6H catalyst are presented in Figure 1b and c, respectively. In catalyst preparation via the impregnation method, metal salts are introduced onto the pores and surface of the carrier. Upon drying, calcination, and decomposition, these salts may occupy some pores, thereby reducing the specific surface area of the catalyst [14]. The specific surface area of the BY-6H catalyst calculated though the BET method was 179 m^2^/g with pore size of 2–54 nm, mainly between 1 and 10 nm, indicating that the catalyst was mainly of mesoporous structure [15].

The TEM characterization results, as shown in Figure 1d,e, indicate that active components uniformly dispersed on composite oxide support. The particle size of the active metal atom clusters ranged from 1.6 to 3.4 nm and the average particle size was calculated to be around 2.36 nm (Figure 1f). Notably, it can be observed that only the lattice fringes of 0.352 nm corresponding to TiO_2_(101) are visible, with no lattice fringes attributed to Al_2_O_3_, indicating good dispersion of TiO_2_ on the surface of the composite oxide support. Lattice fringes corresponding to Ni_2_O_3_(101), MoO_3_(021), MoO_3_(120), and NiMoO_4_ (220) are observed, whereas no fringes corresponding to NiO can be seen. Through the TEM images and particle size distribution, it can be seen that the particles were highly dispersed on the Al_2_O_3_–TiO_2_ support with small particles.

**Figure 2:**
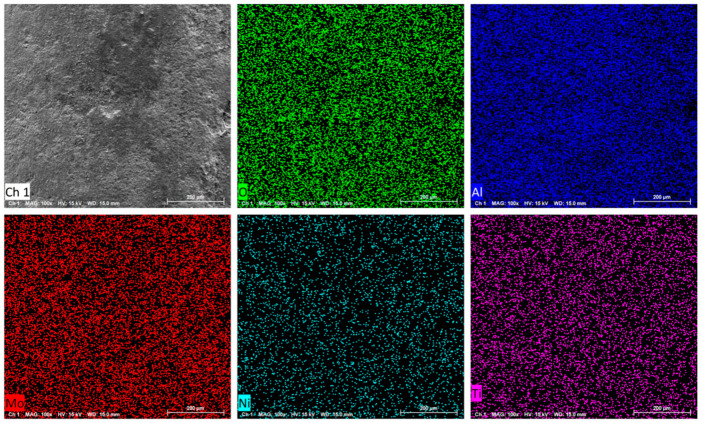
SEM images and the corresponding elemental mappings of BY-6H catalyst.

Figure S4 presents the SEM images of the support. It can be observed that TiO_2_ dispersed well on the surface of Al_2_O_3_ within the support and thus hindered the lattice fringes of Al_2_O_3_ in the HRTEM image (Figure 1e). Figure S4c,d are the SEM images of the BY-6H catalyst. It can be seen that the active components are uniformly distributed on the surface of the support. Figure 2 illustrates the mapping results of the catalyst. It can be observed that the active metals Mo and Ni on the surface of the catalyst are distributed very uniformly, and the distribution of the active components is highly consistent with the distribution of Ti.

**Figure 3:**
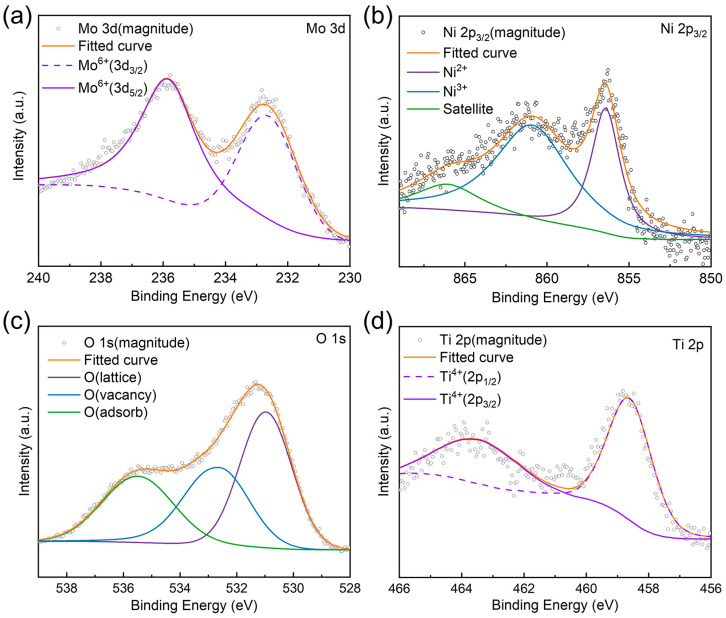
XPS spectra in (**a**) Mo 3d_5/2_, (**b**) Ni 2p_3/2_, (**c**) O 1s, and (**d**) Ti 2p_3/2_ regions of BY-6H.

In Figure 3, Ti 2p binding energy in the BY-6H catalyst shows a peak located at 458.6 eV (2p_3/2_), while the theoretical binding energy for Ti^4+^ is 459.0 eV [16,17,18]. This indicates a reduction in the valence state of Ti in the catalyst, likely due to the influence of nickel (Ni). The Mo 3d binding energy is observed at 235.6 eV, which corresponds to the theoretical binding energy of MoO_3_ (3d_5/2_), suggesting that Mo existed in the form of MoO_3_ [19]. Additionally, compared with the binding energy of Ni_2_O_3_ (855.7 eV), the binding energy (860.1 eV) of Ni 2p_3/2_ in BY-6H moves towards the higher binding energy, indicating that the valence state of Ni increased [20,21]. Consequently, this shift enhances the electron deficiency of Mo and Ni atoms, strengthening the adsorption capacity of active sites for electron-rich olefins, which improves hydrogenation activity and selectivity. Interaction between Mo, Ni, and the TiO_2_ support result in a metal–support interaction that promotes high dispersion of the active metals on the carrier surface, enhancing catalytic performance.

### 2.2. Characterization of the Surface Properties

**Figure 4:**
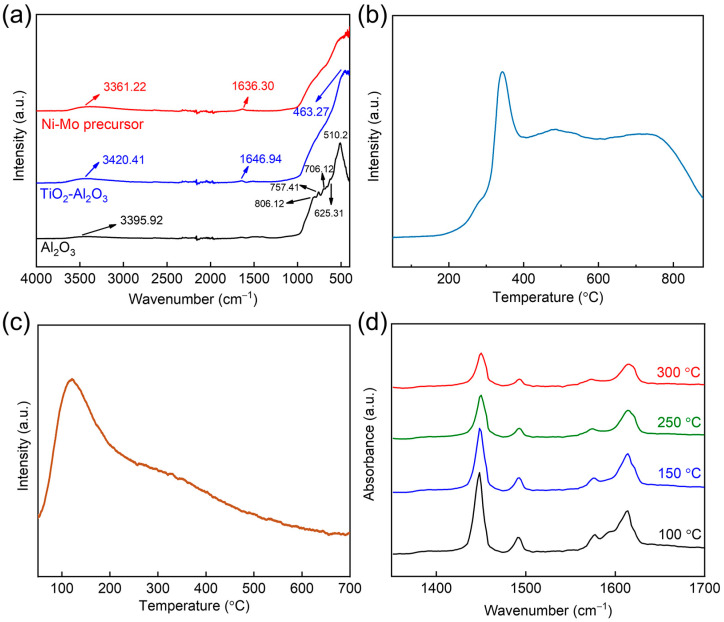
(**a**) FTIR spectra, (**b**) H_2_-TPR profile, (**c**) NH_3_-TPD curve and (**d**) FT-IR spectra of adsorbed pyridine.

Figure 4a displays the attenuated total reflectance infrared (ATR-IR) spectra of the γ-Al_2_O_3_, TiO_2_–Al_2_O_3_, and BY-6H catalysts. In the γ-Al_2_O_3_ spectrum, a peak at 3395 cm^−1^ is assigned to the vibration of hydroxyl groups, while peaks at 806, 757, 706, 625, and 510 cm^−1^ correspond to the characteristic bands of γ-Al_2_O_3_ [22,23]. The spectrum of TiO_2_–Al_2_O_3_ shows a hydroxyl vibration peak at 3420 cm^−1^ [24], a peak at 1646 cm^−1^ corresponding to the vibration of hydroxyl groups in liquid water, and a peak at 1463 cm^−1^ characteristic of γ-Al_2_O_3_. The intensity of characteristic peaks of Al_2_O_3_ at 806, 757, 706, 625, and 510 cm^−1^ have significantly reduced upon the incorporation of Ti, indicating that Ti weakens the coordination between Al and O, leading to the emergence of Al–O–Ti vibrations [17]. The introduction of TiO_2_ in the composite support reduces the strong interactions between active metals such as Mo and Ni with Al, which is beneficial for enhancing the catalyst’s activity. This observation aligns with the XPS analysis and subsequent experimental findings. Consequently, the ATR-IR spectrum of BY-6H shows a peak at 3361 cm^−1^ due to the vibration of hydroxyl groups, a peak at 1630 cm^−1^ corresponding to the vibration of hydroxyl groups in liquid water, and a peak at 1463 cm-1 characteristic of γ-Al_2_O_3_.The strong absorption peaks of OH and H_2_O indicate that a high concentration of Brønsted acid sites is distributed on the support (Figure S5).

H_2_-TPR characterization was further employed to investigate the effect of the composite support on the reducibility of metal species within the catalyst. As shown in Figure 4b, the Mo–Ni/TiO_2_–Al_2_O_3_ catalyst requires a broad temperature range for hydrogen consumption. The low-temperature reduction peak appearing at 300–400 °C corresponds to the reduction in Ni species, while the medium- to high-temperature reduction peak between 400–500 °C is attributed to the reduction in octahedrally coordinated Mo species [25]. A high-temperature reduction peak between 700 and 800 °C is associated with the continuous reduction in tetrahedrally coordinated Mo species or the bulk MoO_3_ phase [26,27,28]. In Figure 4c, it is evident that the catalyst mainly possesses medium-strength and weak acid sites, with minimal strong acidity [29,30]. It is well known that excessively strong acidity can result in coking and carbon deposition on the catalyst surface, which will shorten the catalyst’s lifespan. Conversely, insufficient acidity can hinder olefin activation, thereby reducing hydrogenation activity. Therefore, the presence of appropriately medium-strength acidity on the catalyst surface optimizes both hydrogenation activity and stability, which is consistent with results from subsequent stability tests.

In Figure 4d, four curves show the infrared spectra obtained after temperature-programmed desorption at 100 °C, 150 °C, 250 °C, and 300 °C. It can be observed that there are significant absorption peaks at 1540 cm^−1^ and 1450 cm^−1^, indicating the presence of both Brønsted and Lewis acid sites on the support, with a predominance of Lewis acid sites at 1450 cm^−1^ [31]. Additionally, as the temperature increases, the absorption peak at 1540 cm^−1^ diminishes, and pyridine desorption is nearly complete by 300 °C. The formation of Brønsted acid sites is due to the presence of surface hydroxyl groups, while Lewis acid sites arise from metal atoms with empty orbitals capable of accepting lone pair electrons. In the TiO_2_–Al_2_O_3_ composite support, the Ti-O-Al bond creates charge imbalance, resulting in the formation of Ti-O(H)-Al bonds, which introduce new Brønsted acid sites. However, the formation of Ti-O(H)-Al requires protons, which can be supplied by OH groups. Therefore, the stronger the OH absorption peaks on the support, the greater the number of Brønsted acid sites on its surface. Aa shown in the IR spectrum in Figure 4a, the strong absorption peaks of OH indicate a relatively high concentration of Brønsted acid sites on the support.

### 2.3. Catalytic Performance Evaluation

The catalytic performance was tested in the fixed bed reactor. The reaction conditions were as follows: reaction pressure: 2.5–3.5 MPa, hydrogen to oil ratio (volume ratio): 200:1 to 300:1, reactor inlet temperature: 90–140 °C, and raw material space velocity: 0.1–0.6 h^−1^. The raw material can be 1-butene (99.9 wt%) or butene/isononyl alcohol (15 wt%). The product was analyzed by an Agilent 7820 GC (See Supplementary Materials for more details).

**Figure 5:**
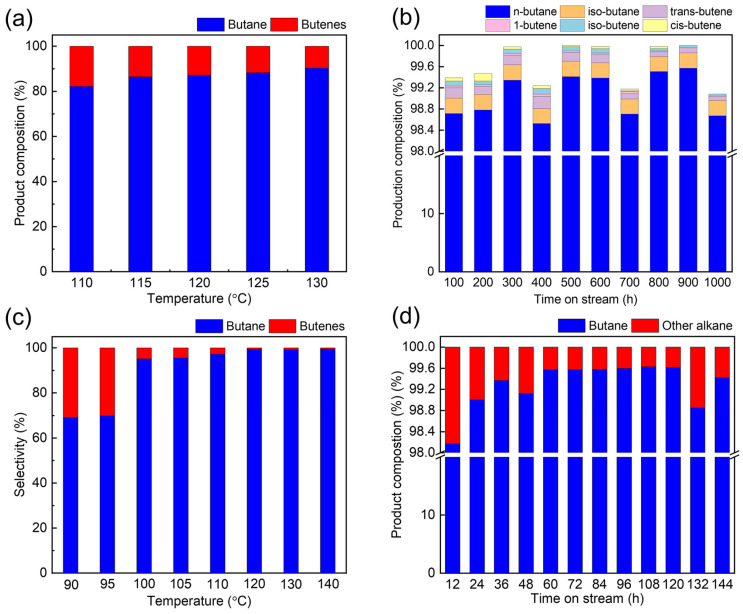
Catalytic performance. (**a**) The variation in olefin saturation rate in products and hydrogenation product composition of C4 olefins at different temperatures with BY-6H catalyst (reaction conditions: 2.5 MPa, 0.6 h^−1^, and 99.9 wt% butene as the feed). (**b**) Hydrogenation product composition of C4 fraction and olefin saturation rate in BY-6H stability experiment (reaction conditions: 2.5 MPa, 125 °C, 0.2 h^−1^, and 99.9 wt% butene as the feed). (**c**) The variation in olefin saturation rate in products and hydrogenation product composition of C4 olefins at different temperatures with BY-6H catalyst (reaction conditions: 3.5 MPa, 0.6 h^−1^, and 99.9 wt% butene as the feed). (**d**) Evaluation of olefin saturation activity under hydrogenation and product composition (industrial simulation, with reaction conditions: 2.5 MPa, 130 °C, 2.0 h^−1^, and 15 wt% butene/isononyl alcohol as the feed).

The selective hydrogenation activity results for the BY-6H catalyst are shown in Figure 5. As seen in Figure 5a, at a pressure of 2.5 MPa and a space velocity of 0.6 h^−1^, the olefin content in the product at 130 °C is 9.7 wt.%, exceeding the industrial requirements for hydrogenated products (olefins < 5%). This suggests that the process conditions require further optimization to achieve the desired hydrogenation level. Figure 5c presents the product analysis for the BY-6H catalyst under high-pressure, low-temperature hydrotreatment conditions (3.5 MPa, 0.6 h^−1^, and a hydrogen-to-oil volume ratio of 300). Utilizing the BY-6H catalyst and under high-pressure conditions, the olefin content in the product at 110 °C was reduced to below 3 wt.%, indicating good low-temperature performance. Traditional hydrogenation of olefins is usually associated with hydrodesulfurization reactions, which often require high reaction temperatures. Thus, low-temperature activity has great influence on the hydrogenation of olefins because high temperatures would cause serious carbon deposition [32,33]. This finding suggests that increasing reactor pressure effectively enhances the low-temperature activity of the BY-6H catalyst. Furthermore, another experiment of hydrotreatment of C_4_/C_5_ fractions was carried out with the BY-6H catalyst at a reaction temperature of 125 °C, and the results are shown in Table S4 and Figure S6. It can be seen that the olefin content in the product was less than 3 wt.%. These results confirm that under the optimized conditions, the catalyst demonstrates effective low-temperature activity and meets the technical specifications outlined in the contract.

The results of the BY-6H catalyst’s low-temperature hydrotreatment stability test for the C_4_ fraction are provided in Table S5 and Figure 5b. The BY-6H catalyst was employed for hydrotreatment of the C_4_ fraction at a reaction temperature of 125 °C. At a reaction time of 1000 h, the 1-butene conversion and n-butane sensitivity were 100% and 98.7%, respectively (Table S5). During a 1000 h stability test, the olefin content in the product remained below 0.4 wt.%, demonstrating good low-temperature activity and stability. These results indicate that the catalyst meets all expected performance criteria, supporting its suitability for industrial application. At the Isooctanol Plant of Maoming Petrochemical, the butene unit includes a 40,000-ton annual capacity hydrogenation unit for the C_4_ fraction, with isononyl C_4_ as the feedstock, containing approximately 15% C_4_ olefins. The hydrogenation reactor is designed with a space velocity of 1 h^−1^, and the hydrogenated product must contain no more than 5 wt.% olefins to meet downstream cracking unit requirements. In this study, butene was used in a simulation test to evaluate the BY-6H catalyst’s suitability for the plant’s operating conditions.

The hydrogenation saturation activity data for the BY-6H catalyst are shown in Figure 5d. After 144 h of reaction, the olefin content in the hydrogenated product was undetectable, indicating that the BY-6H catalyst’s olefin saturation performance meets the technical requirements. Under simulated conditions with a space velocity of 2 h^−1^, using isononyl C_4_ feedstock containing 15 wt.% olefins, an inlet temperature of 130 °C, and a hydrogen-to-catalyst ratio of 160:1, the olefin saturation activity of BY-6H was evaluated. Even when the feed space velocity was doubled, the stabilized olefin saturation activity remained at 100%, confirming that the catalyst BY-6 exhibits excellent hydrogenation saturation performance. This meets the industrial application standards required for isononyl C_4_ hydrogenation at Maoming Petrochemical. For C_4_/C_5_ hydrotreatment, the catalysts mostused in the industry are Pd-based catalyst. For a catalyst with Pd loading of 0.3 wt%, the cost of Pd alone can reach USD 100,000/ton of catalyst. For Ni–Mo-based catalysts, this can be just USD 25,000–35,000/ton of catalyst in total. The low cost of Ni–Mo-based catalysts can have a positive effect on the petrochemical industry.

## 3. Conclusions

This study successfully developed a BY-6H low-temperature hydrotreatment catalyst and evaluated its performance under various conditions, confirming its effectiveness in converting olefins in C_4_ and C_5_ fractions to saturated hydrocarbons. Operating at 2.5 MPa and 130 °C, with a hydrogen-to-oil ratio of 160:1, the BY-6H catalyst achieved undetectable olefin levels. At a space velocity of 2 h^−1^, it maintained over 100% olefin saturation activity, even under doubled feed conditions. In a 1000 h stability test, BY-6H sustained its performance, showing reliability for industrial applications. In trials at Maoming Petrochemical, it met technical standards, highlighting its potential for industrial use. This development offers an efficient, eco-friendly solution for C_4_/C_5_ hydrotreatment, bringing new value to the petrochemical industry.

## Data Availability

The original contributions presented in the study are included in the article/Supplementary Material, further inquiries can be directed to the corresponding author.

## Supplementary Materials

The following supporting information can be downloaded at: https://www.mdpi.com/article/10.3390/nano14231934/s1.
