# Supplementary material for: Low-Temperature Hydrotreatment of C4/C5 Fractions Using a Dual-Metal-Loaded Composite Oxide Catalyst
Source: Nanomaterials (Basel). 2024 Nov 30;14(23):1934. doi: 10.3390/nano14231934 (PMC11643592; doi:10.3390/nano14231934)
Supplement: Supplementary file 1 [file nanomaterials-14-01934-s001.zip › nanomaterials-3303665-supplementary.pdf]

Supporting Information

Low-temperature hydrotreatment of C4/C5 fractions using a dual-metal-loaded composite oxide catalyst

Zhou Du[1,2], Renyi Li[1], Zhenghui Shen[1], Xiao Hai[1] and Ruqiang Zou[1,*]

[1]*Beijing Key Laboratory for Theory and Technology of Advanced Battery Materials, School of Materials Science and Engineering, Peking University, No. 5 Yiheyuan Road, Haidian District, Beijing 100871, China*
[2]*Yanshan Branch, SINOPEC (Beijing) Research Institute of Chemical Industry Co., Ltd., No.15 Fenghuangting Road, Fangshan District, Beijing 102500, China*

Characterization Methods of Catalysts

X-Ray Diffractometry (XRD)

XRD was used for phase analysis of the carrier and catalyst samples using an Empyrean-type polycrystalline powder diffractometer produced by the Dutch Panalytical company, with a Cu Kα radiation source, PIX cel3D detector, tube voltage of 40 kV, tube current of 40 mA, step angle of 0.02°, scanning speed of 2° per minute, and a 2θ scanning range of 10° to 90°.

Low-temperature N_2 Physical Adsorption–Desorption Tester (BET)

An ASAP 2020-type automatic physical and chemical adsorption analyzer from Micromeritics of the United States was used for a full analysis of the micro-meso pore adsorption isotherm. The specific surface area was calculated based on the BET model, and the pore size was calculated based on the BJH model. Analysis conditions comprised nitrogen as the adsorbate, adsorption temperature of –195.8°C, and He gas as the carrier gas. Specific surface area, pore volume, and most probable pore diameter of the sample were determined using the static volumetric method.

Inductively Coupled Plasma (ICP) Spectroscopy

An Optima ICP 7300 V inductively coupled plasma atomic emission spectrometer was used to determine the content of the active component palladium in the catalyst,

with Ar as the working gas. The principle is that various substances emit spectral lines of different wavelengths when excited. Analysis conditions: 100 mg of the sample was dissolved in aqua regia and heated to promote dissolution, then transferred to a volumetric flask, the concentration of the component to be detected was diluted to 1–10 mg/L, and the element measurement was started.

Temperature-Programmed Reduction (TPR) Tester

An AutoChem II 2920-type temperature-programmed adsorption instrument produced by Micromeritics was used for hydrogen temperature-programmed reduction (H_2-TPR) testing of the catalyst sample. Analysis conditions: the catalyst sample was flushed with He gas at 200°C for 1 hour, cooled to –20 °C, the gas was switched to a 10% H_2–Ar mixed gas, then heated at a rate of 10°C/min to 900 °C, and the signal of consumed hydrogen was recorded with a TCD detector.

Scanning Electron Microscopy (SEM)

A Quanta 200-type electron microscope from FEI of the United States was used to observe the morphology and size of the sample crystals, with a magnification of up to 50,000 times.

X-Ray Photoelectron Spectroscopy (XPS)

A Sigma Probe electron spectrometer produced by Thermo VG Scientific of the United Kingdom was used to determine the relative content of different elements on the catalyst surface and the relative content of different electron valence states of the same element. The vacuum in the analysis chamber was less than 10-6 Pa, with Al Kα as the excitation source (Al Kα = 1486.6 eV), and C1s (284.6 eV) was used as an internal standard to calibrate the electron-binding energy of the measured elements.

Ammonia Temperature-Programmed Desorption (NH_3-TPD)

A chemical adsorption instrument, AutoChem 2920 from the American Mac Instrument Company, was used, with a powder sample loading of 0.01 g. It was first flushed with flowing nitrogen at 600 °C for 1 hour, then cooled to 50 °C. Ammonia

was introduced for adsorption. After saturation, nitrogen was flushed until the baseline was stable, and the temperature was raised from 50 °C at a rate of 10 °C/min to 700 °C/min. The ammonia temperature-programmed desorption signal was recorded with chromatography to examine the surface acid amount and acid distribution of each catalyst.

Infrared (IR) Analysis

A Nicolet is50 Fourier transform infrared spectrometer produced by the American Nicolet Company was used to characterize the sample, with a resolution of 4 cm^{-1}, 32 scans, and a scanning range of 4000 to 400cm^{-1}.

High-Resolution Transmission Electron Microscopy (HR-TEM) Analysis

A high-resolution transmission electron microscope (Jem-3010) was utilized to observe the catalyst, with an accelerating voltage of 200 kV for HR-TEM characterization. Prior to testing, the sample was thoroughly ground into a powder. A small amount of the sample was then added to anhydrous ethanol and placed in an ultrasonic cleaner (model PS-10, Shenzhen Jie Kang Ultrasonic Cleaning Machine Co., Ltd.) for ultrasonic separation for 15 to 20 minutes. Finally, the suspension was applied continuously onto a microgrid using a pipette and allowed to air-dry.

Evaluation Methods of catalysts

A flowchart of the hydrogenation catalyst evaluation setup is shown in **Figure S2**, and the actual setup is shown in **Figure S3**. The experimental steps are as follows. Take a certain mass of the catalyst sample and load it into the reaction tube. After testing and ensuring the airtightness of the setup, continuously introduce a hydrogen stream into the reaction tube and gradually heat the tube to the catalyst's sulfurization temperature. Introduce the sulfurization agent and maintain it at sulfurization temperature for a period, then cool to room temperature. Adjust the catalyst bed to the reaction temperature, use a back pressure valve and open the hydrogen bypass to set the system pressure to the desired reaction pressure, and use a double-plunger pump to inject C4 and C5 raw materials into the catalyst bed at a certain flow rate ratio to initiate the

reaction. The gaseous products are sampled and analyzed for composition by gas chromatography.

Catalyst sulfurization pretreatment: Under conditions of 2.5 MPa pressure and 250°C temperature, with a hydrogen flow rate of 5–20 L/h, use hexane with a CS_2 concentration of 1.5 wt% as the sulfurization oil, passing through the catalyst bed at a rate of 20–50 mL/h. The catalyst loading is 20–50 mL. After 30 hours, the catalyst sulfurization process is completed. Then, adjust the pressure and temperature to the experimental conditions and proceed with the catalyst evaluation. After the reaction becomes stable, take samples for analysis every 12–24 hours.

Experimental conditions:

Reaction pressure: 2.5–3.5 MPa

Hydrogen to oil ratio (volume ratio): 200:1 to 300:1

Reactor inlet temperature: 90–140°C

Raw material space velocity: 0.1–0.6 h^{-1}

Specifications and Sources of Experimental Materials

The experimental chemical materials used in this paper are listed in Table S1, the reaction materials are listed in Table S2, and the experimental equipment is listed in Table S1.

Table S1 Experimental Chemicals

Name	Molecular Formula	Molecular Weight	Specification	Manufacturer
Anhydrous Ethanol	C_2H_6O	46.07	Analytically Pure	Sinopharm Group
Nickel Nitrate	$Ni(NO_3)_2·6H_2O$	290.80	Analytically Pure	Tianjin Jiangtian Chemical
Ammonium Molybdate	$(NH_4)_6Mo_7O_{24}·4H2O$	1235.85	Analytically Pure	Sinopharm Group
Cyclohexane	C_6H_{12}	84.16	Analytically Pure	Sinopharm Group

Carbon Di sulfide	CS_2	76.14	Analytically Pure	Sinopharm Group
Aluminum Oxide Powder	γ-Al_2O_3	101.96		Yantai Henghui Chemical Co., Ltd.
Alumina-Titania Support	TiO_2-Al_2O_3	——	φ3 Clover Leaf	Self-made by Yanshan Branch Institute
Hydrogen	H_2	2.016	99.9%	Yanshan petrochemical
Deionized Water	H_2O	18.01	Analytically Pure	Self-made by Yanshan Branch Institute

Table S2 Reaction Materials

Material	Composition	Manufacturer
Pure Butene	99.9% C_4H_8	Aladdin Co., Ltd.
Raffinate C5	Pentane 52.38%, pentene 46.76%, pentadiene 0.81%	Yanshan Petrochemical

Table S3 Experimental Equipment

Name	Model	Manufacturer
Electric Blast Drying Oven	SY101BS-2	Tianjin Sanshui Science and Technology
Box Resistance Furnace	SX2-10-12	Tianjin Zhonghuan Electric Furnace
Ultrasonic Cleaner	KQ218	Kunshan Ultrasonic Instruments
Double Piston Pump	2ZB-1L	Shenzhou Microscience
Electronic Balance	ME204E	Mettler
Fixed Bed Reaction Apparatus	OLKY-10	Oulu Science and Technology
Gas Chromatograph	7820	Agilent
Electric Heating Jacket	SZCL-2	Shanghai Yingdi

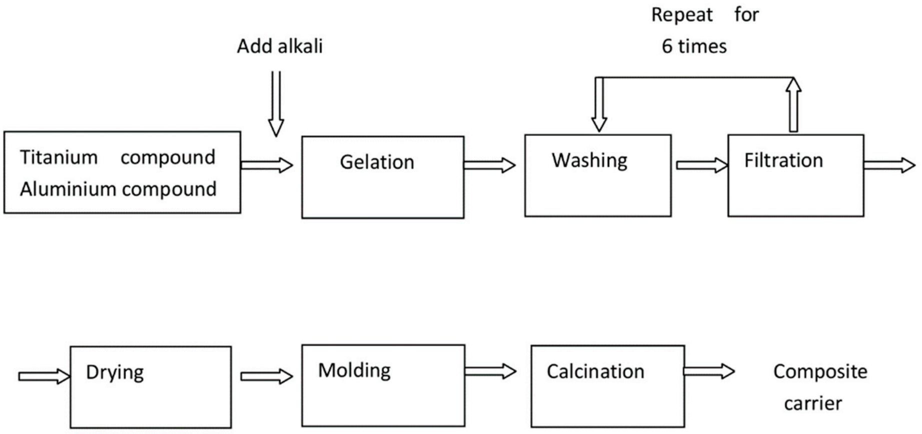

Figure S1 Preparation process of Al_2O_3–TiO_2 composite support.

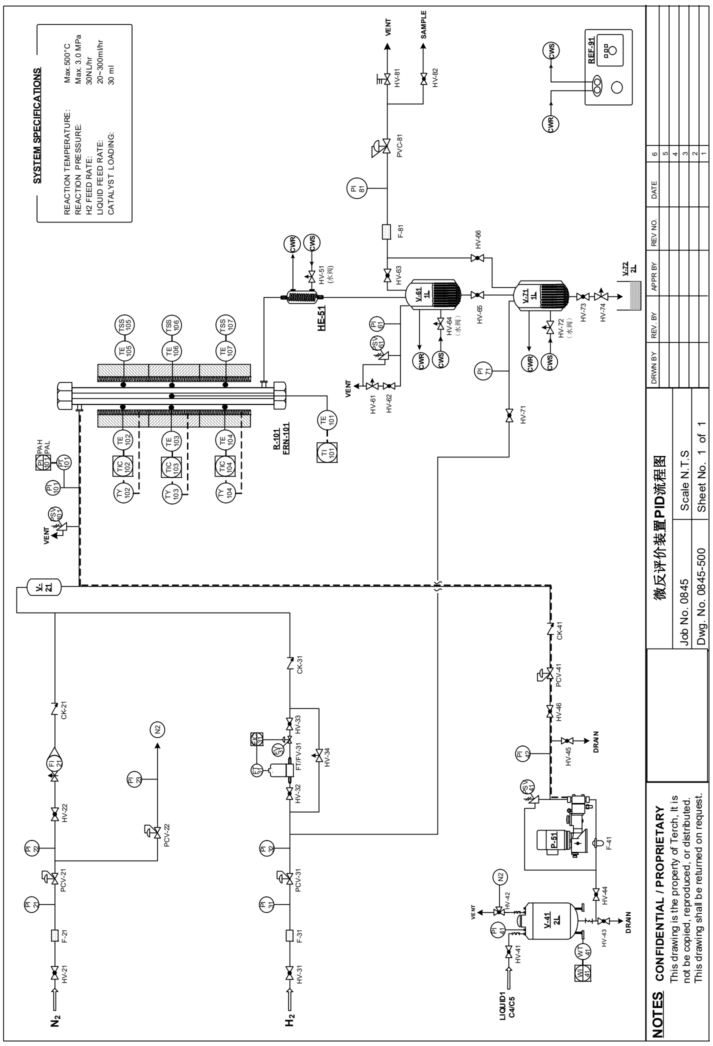

Figure S2 Flowchart of fixed bed evaluation device for hydrogenation catalyst

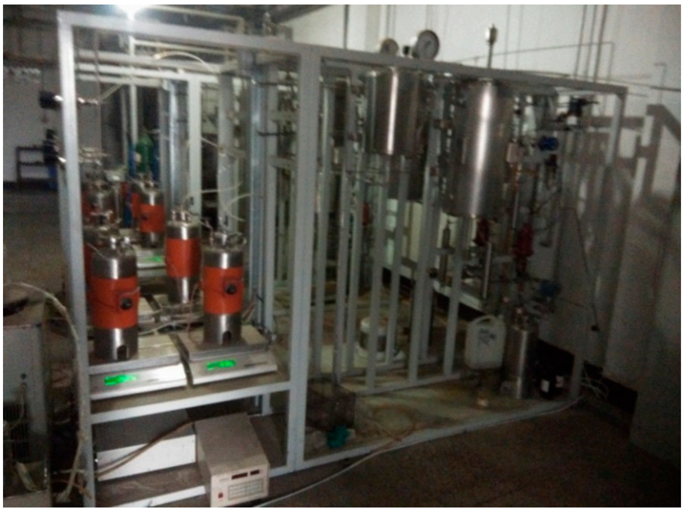

Figure S3 Fixed bed evaluation device for hydrogenation catalyst

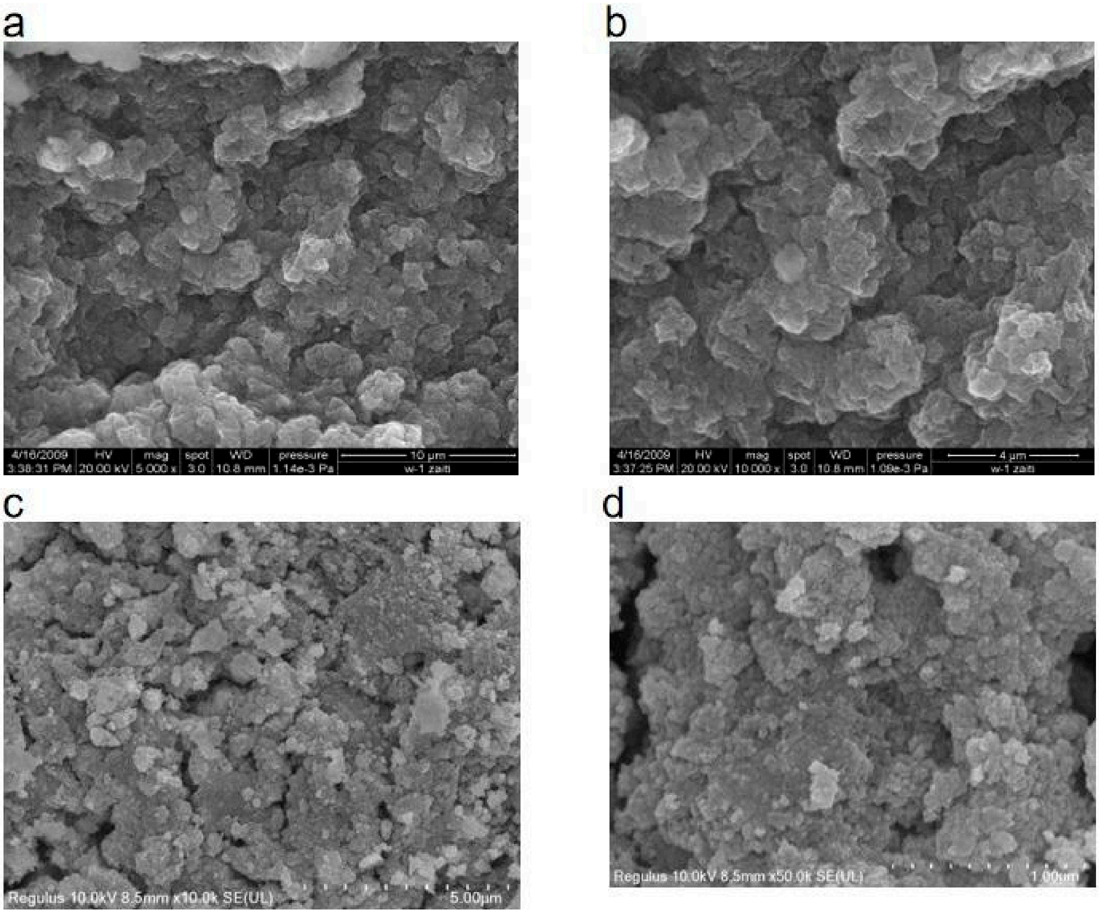

Figure S4. SEM images of **(a)** support (10 μm), **(b)** support (4 μm), **(c)** BY-6H (5 μm) and **(d)** BY-6H (1 μm).

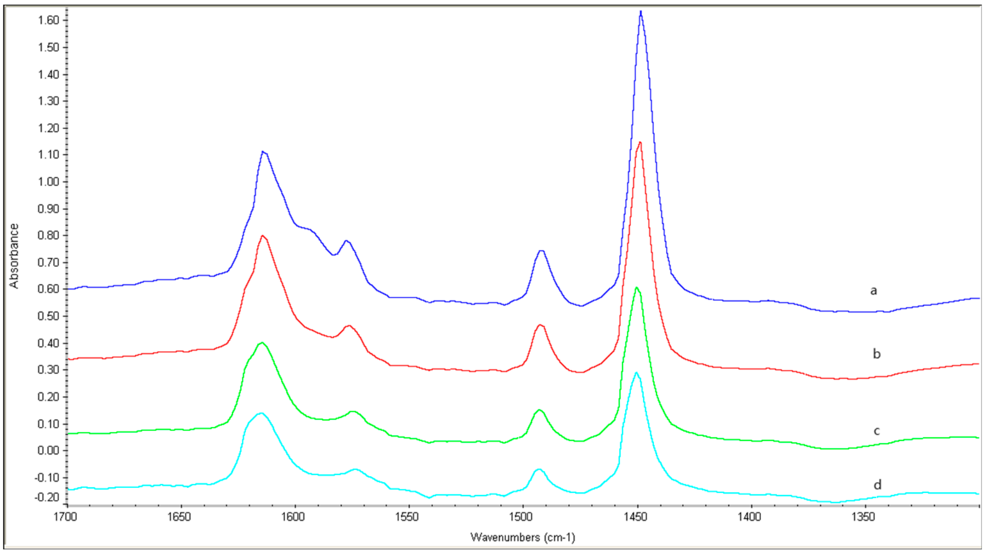

Figure S5 Pyridine adsorption infrared spectra of the support (curves a, b, c, and d show the infrared spectra obtained after the carrier adsorbed pyridine for 30 minutes and was then desorbed at 100 °C, 150 °C, 250 °C, and 300 °C, respectively).

Pyridine, an organic base, can interact with acid sites on solid acid catalysts. Numerous studies reported in the literature have focused on the infrared spectroscopy of pyridine adsorption. A band around 1540 cm^{-1} is characteristic of pyridine adsorbed on protonic acid (Brønsted acid, B acid) sites, while a band around 1450 cm^{-1} is characteristic of pyridine adsorbed on non-protonic acid (Lewis acid, L acid) sites. An absorption band at 1490 cm^{-1} is attributed to the combined effect of pyridine adsorbed on both B and L acid sites. In **Figure S5**, curve a, b, c, and d show the infrared spectra obtained after the carrier adsorbed pyridine for 30 minutes and was then desorbed at 100 °C, 150 °C, 250 °C, and 300 °C, respectively. It can be observed that there are significant absorption peaks at 1570 cm^{-1} and 1450 cm^{-1}, indicating the presence of both B and L acid sites on the support, with a predominance of L acid sites at 1450 cm^{-1}. Additionally, as the temperature increases, the absorption peak at 1570 cm^{-1} decreases, and the pyridine desorption is essentially complete at 300 °C. The formation of B acid sites is due to the presence of surface hydroxyl groups, while L acid sites are formed due to the presence of metal atoms with empty orbitals that can accept lone pair electrons. In the TiO_2–Al_2O_3 composite support, the Ti-O-Al bond, due to charge imbalance, leads to the formation of Ti-O(H)-Al bonds, which are new B acid sites. However, the formation of Ti-O(H)-Al requires protons, which both OH and H_2O can

provide. Therefore, the stronger the absorption peaks of OH and H_2O on the support, the more B acid sites are formed on its surface. From the IR spectra in **Figure S5**, it can be seen that the absorption peaks of OH and H_2O are very strong, indicating that the support has a relatively high concentration of B acid sites.

Table S4 Hydrotreated Product Composition of C4/C5 Fractions over BY-6H

Hydrotreated Product Composition (wt%)				Total
Butane	Pentane	Butenes	Pentenes	olefin(wt%)
62.55	34.781	0.209	2.455	2.664

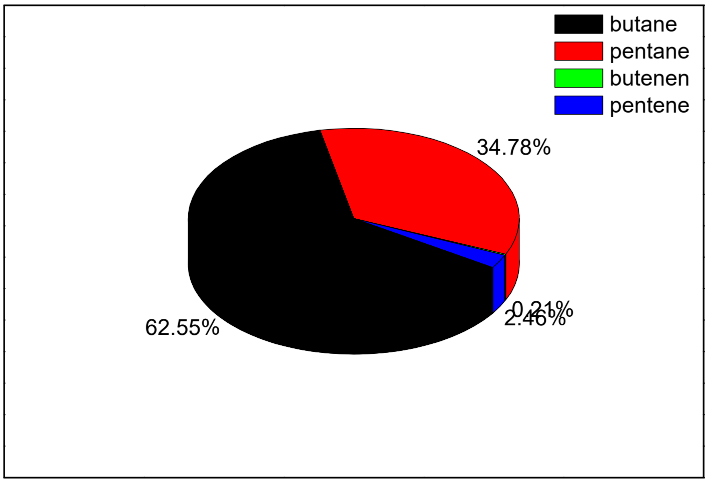

Figure S6. Hydrotreated product composition of C_4 and C_5 Fractions with BY-6H Catalyst (experimental conditions: reaction pressure 2.5 MPa, hydrogen-to-oil volume ratio 3001, reactor inlet temperature 125°C, raffinate C_5 space velocity 0.1 h^{-1}, butene space velocity 0.1 h^{-1}).

Table S5 Hydrotreated product composition of C_4 fraction in BY-6H stability experiment

Operation Time (h)	Hydrotreated Product Composition (wt%)					
	Iso-butane	n-Butane	trans-Butene	cis-Butene	Iso-butene	1-Butene
100	0.289	98.715	0.195	0.034	0.094	0.065
200	0.295	98.781	0.153	0.027	0.073	0.143
300	0.29	99.344	0.18	0.031	0.085	0.049
400	0.281	98.526	0.232	0.039	0.109	0.054

500	0.293	99.410	0.168	0.028	0.080	0.021
600	0.287	99.387	0.161	0.026	0.078	0.042
700	0.285	98.704	0.098	0.041	0.015	0.035
800	0.281	99.506	0.094	0.015	0.046	0.040
900	0.291	99.572	0.097	0	0.04	0
1000	0.287	98.672	0.085	0	0.039	0

Reaction conditions: reaction pressure 2.5 MPa, hydrogen-to-oil volume ratio 2001, reactor inlet temperature 125°C, butene space velocity 0.2 h^{-1}.
